# Screen Time and Standardized Academic Achievement Tests in Elementary School

**DOI:** 10.1001/jamanetworkopen.2025.37092

**Published:** 2025-10-10

**Authors:** Xuedi Li, Charles D. Keown-Stoneman, Jessica A. Omand, Katherine T. Cost, Kelly Gallagher-Mackay, Jennifer Hove, Magdalena Janus, Daphne J. Korczak, Eleanor M. Pullenayegum, Kimberley C. Tsujimoto, Leigh M. Vanderloo, Jonathon L. Maguire, Catherine S. Birken

**Affiliations:** 1 Child Health Evaluative Sciences, The Hospital for Sick Children, Toronto, Ontario, Canada; 2 Li Ka Shing Knowledge Institute, St. Michael’s Hospital, Unity Health Toronto, Toronto, Ontario, Canada; 3 Division of Biostatistics, Dalla Lana School of Public Health, University of Toronto, Toronto, Ontario, Canada; 4 School of Nutrition, Toronto Metropolitan University, Toronto, Ontario, Canada; 5 Department of Psychiatry and Behavioural Neurosciences, Faculty of Health Sciences, McMaster University, Hamilton, Ontario, Canada; 6 Offord Centre for Child Studies, Faculty of Health Sciences, McMaster University, Hamilton, Ontario, Canada; 7 Department of Law and Society, Faculty of Liberal Arts, Wilfrid Laurier University, Waterloo, Ontario, Canada; 8 The Education Quality and Accountability Office, Government of Ontario, Toronto, Ontario, Canada; 9 Neurosciences and Mental Health, The Hospital for Sick Children, Toronto, Ontario, Canada; 10 Department of Psychiatry, Temerty Faculty of Medicine, University of Toronto, Toronto, Ontario, Canada; 11 ParticipACTION, Toronto, Ontario, Canada; 12 School of Occupational Therapy, Western University, London, Ontario, Canada; 13 Department of Pediatrics, Temerty Faculty of Medicine, University of Toronto, Toronto, Ontario, Canada; 14 Institute of Health Policy, Management and Evaluation, Dalla Lana School of Public Health, University of Toronto, Toronto, Ontario, Canada; 15 Department of Nutritional Sciences, Temerty Faculty of Medicine, University of Toronto, Toronto, Ontario, Canada; 16 Department of Pediatrics, St Michael’s Hospital, Unity Health Toronto, Toronto, Ontario, Canada; 17 Division of Pediatric Medicine, The Hospital for Sick Children, Toronto, Ontario, Canada

## Abstract

**Importance:**

Few studies have investigated the longitudinal associations between different types of screen time in young children and academic achievement in elementary school.

**Objective:**

To examine whether there is an association between screen time in young children and standardized academic achievement tests in grades 3 and 6.

**Design, Setting, and Participants:**

This prospective cohort study was conducted among children participating in the TARGet Kids! primary care cohort in Ontario, Canada, between July 2008 and June 2023. Participant data were linked to annual grades 3 and 6 provincial standardized academic achievement test results.

**Exposures:**

Parent-reported child total screen time, TV and digital media time, and video gaming time. The screen time measurement closest before the outcome was used.

**Main Outcomes and Measures:**

Academic achievement levels on standardized tests in reading, writing, and math for grades 3 and 6 were classified as below, at, or above the Ontario provincial standard.

**Results:**

This study included 3322 grade 3 children (mean [SD] age at test, 8.86 [0.28] years; 1714 [51.6%] male students) and 2084 grade 6 children (mean [SD] age at test, 11.86 [0.28] years; 1070 [51.3%] male students). Screen time was measured at mean (SD) age of 5.54 (2.36) years for grade 3 children and 7.54 (2.90) years for grade 6 children. From adjusted proportional odds models, each additional hour of total screen time was associated with 9% to 10% lower odds of achieving a higher academic level in grade 3 reading (odds ratio [OR], 0.91; 95% CI, 0.86-0.96; *P* = .001), grade 3 math (OR, 0.91; 95% CI, 0.86-0.96; *P* < .001), and grade 6 math (OR, 0.90; 95% CI, 0.84-0.96; *P* = .002). Similarly, higher TV and digital media time was associated with lower achievement levels in grade 3 reading and math and grade 6 math. Video game use was associated with lower achievement level in grade 3 reading (OR, 0.77; 95% CI, 0.62-0.94; *P* = .01). In the sex-stratified analysis, video game use among female students was associated with lower grade 3 reading and math achievement.

**Conclusions and Relevance:**

In this prospective cohort study of Canadian children recruited from primary care settings, high levels of total screen time and TV and digital media in young children were associated with lower achievement levels in reading and math on standardized tests in elementary school. Early interventions to reduce screen time exposure should be developed and tested to enhance academic achievement in elementary school.

## Key Points

**Question:**

Is there an association between different types of screen time in young children and academic achievement in grades 3 and 6, as measured by standardized tests in reading, writing, and math?

**Findings:**

In this cohort study of 3322 grade 3 children and 2084 grade 6 children recruited from primary care settings in Ontario, Canada, between 2008 and 2023, higher parent-reported total screen time and TV and digital media time were associated with lower reading and math achievement on standardized tests in elementary school.

**Meaning:**

These findings suggest that early interventions to reduce screen time exposure should be developed and tested to promote healthy screen use habits and enhance academic achievement in elementary school.

## Teaser

This cohort study evaluates whether there is an association between different types of screen time, including TV, digital media, and video gaming, and standardized academic achievement test results in reading, writing, and math in grades 3 and 6 among children in Canada.

## Introduction

With digital media devices ubiquitous in daily life, children are exposed to high levels of screen time from a young age, despite US and Canadian pediatric recommendations to limit recreational screen time for young children.^1,2,3,4,5^ Total screen time, capturing all screen-based activities, is a critical measure of health behavior in children.^1^ It is also essential to consider the types of screen time, as not all screen time presents the same risks and benefits.^6^ While high-quality screen time, such as interactive and educational content that is coviewed or coplayed with parents, can offer potential benefits for children’s learning and development,^7^ high levels of screen time remain a significant concern, given evidence linking it to negative health, mental health, and education outcomes in children.^8,9,10,11^

Academic achievement is an important indicator of education success and is linked to later health and educational outcomes.^12,13^ It is measured in a variety of ways, including school grades, grade point average, and performance on standardized tests for main subject areas including reading, writing, and math.^11,14^ Child screen time may be linked to academic achievement in several ways. Screen time may displace academic-promoting activities, such as physical activity, peer play time, and sleep.^15,16^ High levels of screen time may alter children’s brain structure, affecting cognitive functions and impairing the acquisition of memories and learning.^17^ The constant distraction from screen time may impede child social development.^17^ Alternately, screen time may enhance academic achievement through increased access to resources and information.^18^ Systematic reviews have demonstrated that TV viewing and video gaming were negatively associated with academic outcomes in children and youth.^11,14^ However, much of the existing literature on screen time and academic achievement have been cross-sectional and focused on children and youth 12 years and older.^19,20,21^ There is a lack of longitudinal studies examining screen time across early-to-middle childhood and on academic achievement in elementary school. It is important to study screen time in young children as screen behavior patterns are established during the early years.^22^ A child’s earliest screen encounters are habit-forming, with patterns of exposure and use often persisting into later life.^1^ Most studies on screens and school outcomes focus on older children and youth, despite evidence of high use in younger children.^7^ Understanding the relationship between early screen time and academic achievement would help identify key targets for preventative strategies and interventions implemented early in the transition to school.

The primary objective of this study was to determine whether total screen time, TV and digital media time, and video game time in young children were associated with standardized academic achievement tests in grades 3 and 6 among children in Ontario, Canada. The secondary objective was to examine these potential associations separately in male and female children, as previous research has demonstrated sex differences in total and different types of screen time^23,24^ and academic achievement in elementary students.^25,26,27^ We hypothesized that higher levels of screen time would be associated with lower academic achievement in all areas, with variation in associations in different types of screen time and by sex.

## Methods

### Study Population and Design

A prospective cohort study was conducted among children aged 0 to 12 years participating in the TARGet Kids! research network^28^ in Ontario, Canada between 2008 and 2023.^29^ TARGet Kids! is an ongoing primary care practice–based cohort with 10 large primary care clinics participating in the greater Toronto area and Kingston, Canada.^29^ Since 2008, we have been enrolling healthy young children aged 0 to 5 years at primary care practices and following up with them into adolescence.^29,30^ TARGet Kids! exclusion criteria include children with chronic conditions (with the exception of asthma) at enrollment, children with severe developmental delay at enrollment, children with gestational age less than 32 weeks, and families who are unable to complete the consent and/or questionnaires in English.^29^ At each well-child visit, parents are invited to complete an age-specific standardized questionnaire with questions on sociodemographic information, child and parent physical and mental health, and health behaviors (eg, screen time, sleep, and physical activity).^29^

Since 2012, TARGet Kids! participating families have provided consent to access their children’s Ontario provincial standardized academic achievement test data through the Ontario Education Quality and Accountability Office (EQAO),^31^ a Crown agency of the Government of Ontario responsible for monitoring the quality and providing accountability for Ontario’s publicly funded kindergarten through grade 12 education system.^31^ Our participant data are linked to children’s standardized academic achievement tests in reading, writing, and math for grade 3 (2012-2023) and grade 6 (2015-2023). Tests were not administered in the 2020 and 2021 academic years due to the COVID-19 pandemic.

This study was approved by the Research Ethics Boards at The Hospital for Sick Children and Unity Health Toronto, and Clinical Trials Ontario. All parents or caregivers of participants provided informed oral or written consent. The Strengthening the Reporting of Observational Studies in Epidemiology (STROBE) reporting guideline for cohort studies has been followed.^32^

### Exposure: Child Screen Time

The primary exposure was child total screen time. Secondary exposures were TV and digital media time and video gaming time. Screen time data have been collected repeatedly through parent-reported questionnaires at various time points since 2008, with questions on the child’s daily time spent on watching TV, watching DVDs, playing on the computer, playing video games, and playing with handheld devices (eg, smartphones, iPads) on a typical day. Child total screen time was calculated as the sum of all screen activities. TV and digital media time was derived from the combined time spent on TV, DVDs, computer, and handheld devices. The most recent screen time measure collected prior to standardized academic achievement tests was used for analysis (ie, the latest screen time data before the grade 3 standardized test served as the exposure for grade 3 analysis, and the latest screen time data before the grade 6 standardized test was used for grade 6 analysis).

### Outcome: Standardized Academic Achievement Tests in Grades 3 and 6

The outcome of this study was standardized academic achievement tests in reading, writing, and math administered by the EQAO for grades 3 and 6. As key indicators of academic achievement, these tests are conducted annually across all Ontario school boards and are grounded in the Ontario curriculum, outlining the knowledge and skills students are expected to acquire in domains of literacy (reading and writing) and math at each grade level. EQAO uses a rigorous test development process and stringent scoring procedures to ensure validity, reliability, accuracy, and consistency in year-to-year assessments.^33^ The test results are reported as levels of achievement, defined by the Ontario Ministry of Education. The achievement level in each subject area of reading, writing, and math ranges from a low level of 1 to a high level of 4, with levels 1 and 2 indicating below the provincial standard, level 3 indicating meeting the standard, and level 4 indicating exceeding the standard. Student achievement information is comparable year over year to track performance over time. In this study, academic achievement levels for grades 3 and 6 were categorized as ordinal: below, at, or above the provincial standard for each subject area.

### Covariates

All covariates were selected a priori from the literature. Potential confounders and factors associated with outcomes included in the models were: child age at standardized test, child sex assigned at birth, maternal education, child ethnicity, self-reported family income, child living arrangement, duration between the exposure and outcome measurements, Individual Education Plan (IEP) status (a written plan that describes special education programs, accommodations and services that a school board will provide for a student), and year of standardized test. Data on sociodemographic covariates were obtained from parent-reported questionnaires. Child ethnicity was assessed in this study as a key sociodemographic variable and a potential confounder in the analysis. Ethnicity data were collected using parent-reported standardized questionnaires, which included 22 detailed categories. For analysis, these categories were consolidated into broader groups: African, Arab, East Asian, European, Latin American, multiethnic, South Asian, Southeast Asian, and Indigenous, Oceania, or other (which included open-text responses, unknown, and prefer not to answer). Child ethnicity was derived from maternal and paternal responses and classified as multiethnic if parents selected more than 1 category or if maternal and paternal ethnicities differed. Information on student IEP status was sourced from EQAO records. The Ontario provincial curriculum and standardized tests have evolved over the years, and screen-based technology has changed significantly in the last decade. This study included data on screen time and standardized tests before and during the COVID-19 pandemic (tests were not administered in 2020 and 2021 due to the pandemic). To account for changes related to the curriculum, technology, and the pandemic, we adjusted for the year of the standardized test as well as the duration between the exposure date and the provincial standardized test date in our model. Given the sex differences in academic achievement^25,26,27^ and screen time,^23,24^ secondary analyses were stratified by child sex.

### Statistical Analysis

Descriptive analyses were performed to describe participant characteristics, screen time, and academic achievement for the total sample as well as for male and female students with grade 3 and/or grade 6 standardized test data. Cumulative logit proportional odds models were used to examine the association between each type of screen time and the ordinal outcome of academic achievement level in each subject area adjusting for covariates. The proportional odds assumption was assessed using the Brant test to ensure it was not violated. Separate analyses were performed for children with grade 3 and grade 6 standardized test data. Effect estimates are reported as odds ratios (ORs). Secondary analyses were conducted stratifying by child sex.

Missing data were present in some covariates, with missingness ranging from 0.6% to 7.4%. We assumed that data were missing at random conditional on the other variables included in the model. Multivariate imputation with chained equations (using 50 imputed datasets) were performed using the mice package in R to reduce bias from missing covariates.^34^ All *P* values were 2-tailed, and a family-wise level of statistical significance was set at α = .05. A Bonferroni-corrected α was set at .017 to account for multiple comparisons across the 2 primary outcomes (reading, writing, and math). Secondary analyses examining associations in male and female students separately were considered exploratory, without correction for multiple comparisons. R version 4.4.1 for Mac (R Project for Statistical Computing) was used for all analyses.

## Results

A total of 3322 grade 3 children (mean [SD] age at test, 8.86 [0.28] years) and 2084 grade 6 children (mean [SD] age at test, 11.86 [0.28] years) with screen time data prior to their standardized test data were included in this study (Figure). Participant characteristics are summarized in Table 1. Among the 3322 children, 1714 (51.6%) were male; 134 (4.4%) reported African ethnicity, 1778 (58.0%) reported European ethnicity, 699 (22.8%) reported being multiethnic, and 221 (7.2%) had South Asian ethnicity. Overall, 1473 (48.8%) reported a family income greater than CAD $150 000 per year (approximately US $108 506). Screen time exposure was measured as a single data point closest to the outcome, with ages varying across participants. For children with grade 3 standardized test data, screen time was measured at a mean (SD) age of 5.54 (2.36) years, with a mean (SD) screen time of 89.28 (72.15) min/d (1.6 [1.3] h/d). For children with grade 6 standardized test data, it was measured at a mean (SD) age of 7.54 (2.90) years, with a mean (SD) screen time of 98.83 (71.55) min/d (1.8 [1.4] h/d) (Table 2). The range of screen time measurements spanned from infancy to approximately 10 years. Male participants had higher screen time compared with female participants, particularly in video game time. We further examined the distribution of video game time across the total sample and by sex. Given that video game time was highly skewed toward zero in each group, we analyzed video game as a binary variable (any vs none) instead of a continuous measure. In grade 3, 1449 children (55.9%) were at the provincial standard for reading, 1999 (77.2%) for writing, and 1788 (53.8%) for math. In grade 6, these figures were 1428 (68.6%) for reading, 1267 (60.9%) for writing, and 1027 (49.3%) for math. In our sample, female students outperformed male students in reading and writing in both grades 3 and 6, with a higher percentage of female students meeting or exceeding the provincial standard. Math achievement was similar in male and female students in both grades.

**Figure.**
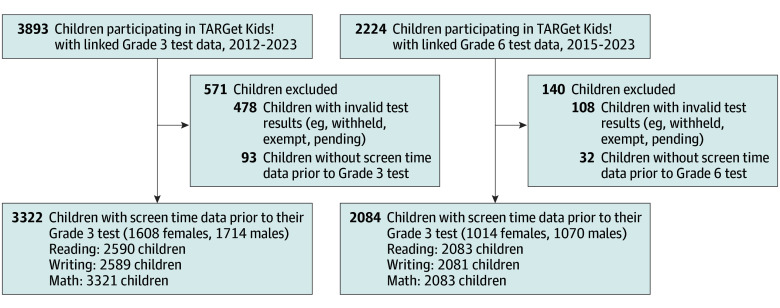
Flowchart of Children Participating in the TARGet Kids! Cohort With Screen Time Data and Provincial Standardized Academic Achievement Test Data, 2008-2023

**Table 1.** Characteristics of Children Participating in the TARGet Kids! Cohort With Screen Time Data and Standardized Academic Standardized Test Data, 2008-2023

Characteristic	Children with grade 3 standardized test data (n = 3322), No. (%)	Missing, %	Children with grade 6 standardized test data (n = 2084), No. (%)	Missing, %
Child age at screen time measurement, mean (SD), y	5.54 (2.36)	0.0	7.54 (2.90)	0.0
Child age at standardized academic achievement test, mean (SD), y	8.86 (0.28)	0.0	11.86 (0.28)	0.0
Child sex				
Female	1608 (48.4)	0.0	1014 (48.7)	0.0
Male	1714 (51.6)		1070 (51.3)	
Maternal education^a^				
College or university	3008 (91.3)	0.8	1874 (90.5)	0.6
High school	251 (7.6)		169 (8.2)	
Public school	36 (1.1)		28 (1.4)	
Child ethnicity^a^				
African	134 (4.4)	7.7	77 (3.9)	5.2
Arab	33 (1.1)		21 (1.1)	
East Asian	103 (3.4)		78 (3.9)	
European	1778 (58.0)		1168 (59.1)	
Latin American	41 (1.3)		32 (1.6)	
Multiethnic	699 (22.8)		452 (22.9)	
South Asian	221 (7.2)		114 (5.8)	
Southeast Asian	55 (1.8)		33 (1.7)	
Indigenous, Oceania, or other	2 (0.1)		1 (0.1)	
Self-reported annual family income, CAD$^a^^,^^b^				
0-39 999	227 (7.5)	9.1	107 (5.5)	7.4
40 000-79 999	424 (14.0)		250 (13.0)	
80 000-149 999	896 (29.7)		556 (28.8)	
≥150 000	1473 (48.8)		1017 (52.7)	
Child living arrangement^a^				
Lives alternating with 2 parents in different households	84 (2.5)	0.0	71 (3.4)	0.0
Lives with 2 parents in the same household	3019 (90.9)		1867 (89.6)	
Other	219 (6.6)		146 (7.0)	
Has Individual Education Plan^c^	419 (12.6)	0.0	546 (26.2)	0.0
Yes				
Year of standardized academic achievement test^d^				
2012	49 (1.5)	0.0	0	0.0
2013	150 (4.5)		0	
2014	298 (9.0)		0	
2015	111 (3.3)		16 (0.8)	
2016	435 (13.1)		116 (5.6)	
2017	471 (14.2)		286 (13.7)	
2018	384 (11.6)		393 (18.9)	
2019	410 (12.3)		494 (23.7)	
2022	537 (16.2)		365 (17.5)	
2023	477 (14.4)		414 (19.9)	

^a^ Sociodemographic data were collected from parent-reported questionnaires. The other category includes open-text responses, unknown, or prefer not to answer. Child ethnicity was derived from maternal and paternal ethnicities and was classified as multiethnic if parents selected more than 1 category or if maternal and paternal ethnicities differed.

^b^ To convert Canadian dollars to US dollars, multiply by 0.72.

^c^ Student Individual Education Plan status data were provided by the Ontario provincial standardized testing organization, the Education Quality and Accountability Office.

^d^ Tests were not administered in 2020 and 2021 academic years due to the COVID-19 pandemic.

**Table 2.** Screen Time and Levels of Academic Achievement Among Children With Grade 3 and/or Grade 6 Standardized Academic Achievement Test Data

Factor	Children with grade 3 standardized test data, No. (%)			Children with grade 6 standardized test data, No. (%)		
	All (n = 3322)	Male (n = 1714)	Female (n = 1608)	All (n = 2084)	Male (n = 1070)	Female (n = 1014)
**Parent-reported child screen time, mean (SD), min/d**						
Total screen time	96.87 (80.46)	101.07 (81.50)	92.38 (79.12)	110.39 (81.06)	116.57 (84.70)	103.87 (76.53)
TV and digital media	89.28 (72.15)	90.86 (71.41)	87.60 (72.92)	98.83 (71.55)	100.48 (73.54)	97.09 (69.37)
Video game	6.61 (20.14)	9.37 (24.44)	3.67 (13.62)	10.12 (25.85)	15.00 (31.20)	4.97 (17.19)
Video game user, No. (%)	686 (20.7)	478 (27.9)	208 (12.9)	561 (26.9)	405 (37.9)	156 (15.4)
**Levels of academic achievement relative to Ontario provincial standard**						
Reading						
Below	336 (13.0)	212 (15.5)	124 (10.2)	140 (6.7)	98 (9.2)	42 (4.1)
At	1449 (55.9)	809 (59.1)	640 (52.4)	1428 (68.6)	771 (72.1)	657 (64.8)
Above	805 (31.1)	348 (25.4)	457 (37.4)	515 (24.7)	200 (18.7)	315 (31.1)
Writing						
Below	414 (16.0)	269 (19.7)	145 (11.9)	163 (7.8)	120 (11.2)	43 (4.2)
At	1999 (77.2)	1036 (75.7)	963 (78.9)	1267 (60.9)	715 (67.0)	552 (54.4)
Above	176 (6.8)	63 (4.6)	113 (9.3)	651 (31.3)	232 (21.7)	419 (41.3)
Math						
Below	764 (23.0)	373 (21.8)	391 (24.3)	696 (33.4)	364 (34.0)	332 (32.8)
At	1788 (53.8)	911 (53.2)	877 (54.6)	1027 (49.3)	516 (48.2)	511 (50.4)
Above	769 (23.2)	430 (25.1)	339 (21.1)	360 (17.3)	190 (17.8)	170 (16.8)

Descriptive results for child screen time by academic achievement levels (below, at, or above the provincial standard) for each subject area in male and female students are provided in eTable 1 and 2 in Supplement 1. Among male students, those with the highest total screen time and TV and digital media time were in the below-standard group, followed by the at-standard group, while those with the lowest screen time were in the above-standard group across both grades in all subject areas. Among female students, similar trends were observed. Among female students across grades 3 and 6 (eTable 2 in Supplement 1), there was a trend that the percentage of video game users was the highest among the below-standard group.

The results of the primary analysis examining the association between screen time and standardized academic achievement tests in the total sample are presented in Table 3. After adjusting for covariates, it was estimated that each additional hour per day of total screen time was associated with 9% to 10% lower odds of achieving a higher academic level in grade 3 reading (OR, 0.91; 95% CI, 0.86-0.96; *P* = .001), grade 3 math (OR, 0.91; 95% CI, 0.86-0.96; *P* < .001), and grade 6 math (OR, 0.90; 95% CI, 0.84-0.96; *P* = .002). There was insufficient evidence of associations with grade 3 writing (OR, 0.94; 95% CI, 0.88-1.01; *P* = .08), grade 6 reading (OR, 0.97; 95% CI, 0.90-1.05; *P* = .45), or grade 6 writing (OR, 0.96; 95% CI, 0.89-1.03; *P* = .21). TV and digital media were similarly associated with lower achievement levels in grade 3 reading and math and grade 6 math (Table 3). There was evidence that video game time (any vs none) was associated with lower achievement level of grade 3 reading (OR, 0.77; 95% CI, 0.62-0.94; *P* = .01), but insufficient evidence was found for associations with the other outcomes. The proportional odds assumption was violated for the model comparing grade 3 writing by video game time (any vs none) model (Brant test *P* = .03). A multinomial regression model was then fitted, and the results of the model presented in Table 3 are based on this model.

**Table 3.** Primary Analysis Results: Association Between Child Screen Time and Levels of Academic Achievement in Reading, Writing, and Math in Grades 3 and 6 Using a Proportional Odds Model^a^

Parent-reported child screen time	Reading^b^		Writing^b^		Math^b^	
	Odds ratio (95% CI)	*P* value	Odds ratio (95% CI)	*P* value	Odds ratio (95% CI)	*P* value
**Children with grade 3 standardized test data (n = 3332)**						
Total screen time, h/d	0.91 (0.86-0.96)	.001	0.94 (0.88-1.01)	.08	0.91 (0.86-0.96)	<.001
TV and digital media, h/d	0.91 (0.85-0.97)	.004	0.93 (0.87-1.01)	.08	0.90 (0.85-0.96)	<.001
Video game, any vs none	0.77 (0.62-0.94)	.01	0.90 (0.67-1.21)^c^	.50	0.85 (0.71-1.01)	.07
**Children with grade 6 standardized test data (n = 2084)**						
Total screen time, h/d	0.97 (0.90-1.05)	.45	0.96 (0.89-1.03)	.21	0.90 (0.84-0.96)	.002
TV and digital media, h/d	0.93 (0.86-1.02)	.11	0.94 (0.87-1.02)	.12	0.89 (0.82-0.96)	.002
Video game, any vs none	1.09 (0.86-1.38)	.47	0.95 (0.76-1.18)	.64	0.99 (0.81-1.22)	.96

^a^ The Bonferroni-corrected α was set at .017 to account for multiple comparisons across the 3 primary outcomes.

^b^ Models were adjusted for child age at test, child sex, child ethnicity, maternal education, self-reported annual family income, child living arrangement, year of test, duration between screen time date and test date, and Individual Education Plan status.

^c^ The proportional odds assumption was violated for this model (*P* = .03). A multinomial regression model was then fitted, and the results presented in the table are based on this model.

Results of the secondary analyses stratified by child sex are provided in Table 4. Both male and female students exhibited similar findings, with higher total screen time and TV and digital media time associated with lower achievement levels in grade 3 reading and math and grade 6 math (Table 4). Video game time (any vs none) was associated with lower achievement levels in grade 3 reading (OR, 0.67; 95% CI, 0.47-0.95; *P* = .02) and math (OR, 0.69; 95% CI, 0.51-0.93; *P* = .02) among female students, but there was no evidence of these associations among male students (grade 3 reading: OR, 0.83; 95% CI, 0.64-1.08; *P* = .16; grade 3 math: OR, 0.97; 95% CI, 0.78-1.22; *P* = .80).

**Table 4.** Secondary Analyses Results: Association Between Child Screen Time and Levels of Academic Achievement in Reading, Writing, and Math in Grades 3 and 6 Using a Proportional Odds Model in Male and Female Students

Parent-reported child screen time	Reading^a^		Writing^a^		Math^a^	
	Odds ratio (95% CI)	*P* value	Odds ratio (95% CI)	*P* value	Odds ratio (95% CI)	*P* value
**Male students with grade 3 standardized test data (n = 1714)**						
Total screen time, h/d	0.91 (0.84-0.99)	.02	0.95 (0.87-1.04)	.30	0.92 (0.86-0.99)	.03
TV and digital media, h/d	0.91 (0.83-1.00)	.04	0.95 (0.86-1.05)	.33	0.91 (0.84-0.98)	.02
Video game, any vs none	0.83 (0.64-1.08)	.16	0.76 (0.56-1.03)	.08	0.97 (0.78-1.22)	.80
**Female students with grade 3 standardized test data (n = 1608)**						
Total screen time, h/d	0.90 (0.83-0.99)	.02	0.94 (0.85-1.05)	.27	0.89 (0.82-0.96)	.003
TV and digital media, h/d	0.91 (0.83-1.00)	.048	0.94 (0.83-1.05)	.26	0.89 (0.82-0.97)	.009
Video game, any vs none	0.67 (0.47-0.95)	.02	0.70 (0.45-1.08)	.10	0.69 (0.51-0.93)	.02
**Male students with grade 6 standardized test data (n = 1070)**						
Total screen time, h/d	1.00 (0.91-1.11)	.96	1.00 (0.91-1.10)	.97	0.91 (0.83-0.99)	.03
TV and digital media, h/d	0.98 (0.87-1.10)	.68	0.98 (0.88-1.10)	.78	0.91 (0.82-1.01)	.07
Video game, any vs none	1.21 (0.89-1.65)	.23	1.13 (0.84-1.51)	.42	1.09 (0.83-1.43)	.51
**Female students with grade 6 standardized test data (n = 1014)**						
Total screen time, h/d	0.93 (0.83-1.04)	.21	0.92 (0.82-1.02)	.10	0.88 (0.80-0.98)	.02
TV and digital media, h/d	0.89 (0.78-1.01)	.08	0.90 (0.80-1.01)	.07	0.86 (0.76-0.96)	.01
Video game, any vs none	0.92 (0.64-1.35)	.68	0.77 (0.54-1.11)	.16	0.84 (0.60-1.20)	.35

^a^ Models were adjusted for child age at test, child ethnicity, maternal education, self-reported annual family income, child living arrangement, year of test, duration between screen time date and test date, and Individual Education Plan status.

## Discussion

In this prospective cohort study of children recruited from primary care settings in Ontario, Canada, between 2008 and 2023, higher parent-reported total screen time and TV and digital media time were associated with lower academic achievement in reading and math in elementary school. In the combined sample, video game use was associated with lower reading achievement in grade 3. Analysis stratified by child sex revealed that video game use in female students was specifically associated with lower reading and math achievement in grade 3. The effect size may have meaningful public health implications, given the high prevalence of screen use in children and the established link between Ontario’s standardized test performance and postsecondary education outcomes.^35^

Screen time in our sample was measured at various ages prior to the outcome, with most measurements collected at young ages: the mean age at which screen time was measured was 5.5 years for children with grade 3 test data and 7.5 years for children with grade 6 test data. Much of the existing research emphasizes digital and social media use in older children and youths, but greater attention is needed on screen behaviors among young children. Screen time in these formative years may influence child development. Our group previously demonstrated that mobile media device use was associated with expressive speech delay in 18-month-old children,^36^ and higher early screen time was associated with increased vulnerability in teacher-reported developmental readiness for school in children aged 4 to 6 years.^37^ Research has also shown a directional association between early screen time and poor developmental outcomes in children aged 24 to 60 months.^38^ These findings, combined with our study results, underscore the importance of testing early prevention and interventions targeting screen time in young children to support better academic achievement outcomes. Research has suggested that academic interventions are more effective when implemented early, with the greatest benefits observed starting in early elementary school.^39^

In this study, total screen time and TV and digital media time were associated with lower reading and math achievement in elementary school. Our findings align with a systematic review of 58 cross-sectional studies in children and adolescents aged 4 to 18 years, which reported that TV viewing was inversely associated with language and math performance.^14^ Similarly, our findings are consistent with results from several large cohort studies among school-aged children.^18,24,40^ While children today engage in diverse forms of screen time, our findings reaffirm that total screen time and TV and digital media time in younger children remain critical measures in media use research and its relationship with academic achievement. There was no evidence that high screen time was associated with writing achievement in our study. High screen time may be more closely linked to reading achievement than writing for several reasons. Reading development relies on early exposure to language-rich interactions and sustained focus, areas that screen time may replace and disrupt. For instance, a home literacy environment rich with print exposure and shared reading experiences has been shown to be associated with greater reading and math achievement later.^41,42,43,44^ Increasing presence of screens prior to school-ages may present a barrier to the development of critical prereading skills due to the interruption to home literacy activities that set children up for success when formal reading instruction begins.^45^ Reading also requires active engagement with texts to build comprehension, inferencing, and critical thinking. These cognitive skills may be more sensitive to the negative effects of high screen time on sustained attention.^46,47^ In contrast, writing may be less susceptible to the effects of screen time on the home literacy environment. Research has shown that while home literacy environment positively impacts transcription skills, such as handwriting fluency and word spelling, it has a limited impact on higher-level writing skills, such as sentence generation or extended text production.^48^

Research examining video gaming and academic achievement among school-aged children has yielded mixed findings. Some studies linked video game time to lower academic achievement,^49,50^ while others found no association or even positive associations.^18,40,51,52^ In our study, sex-stratified analyses indicated that video game use in female children was specifically associated with lower reading and math achievement in grade 3, while no such association was observed in male children or in grade 6 for either sex. Given the young age of our sample, most children did not engage in video gaming at the age screen time was measured, which may have limited our power to detect associations. Research has shown gender differences in video gaming: video games are more likely to interest boys than girls, and boys and girls have different levels of investment in different types of games.^53^ Potential explanations for this difference may include cognitive development, social and cultural stereotypes, and parental expectations shaped by sex or gender.^54,55^ Due to the young age of the participants in our cohort, data on child gender was not collected, and cannot be explored. Moreover, we lacked detailed and comprehensive information on video game characteristics, such as content, context (eg, violent vs nonviolent, interactive vs passive, educational vs entertainment), and addiction. Further research is needed to explore the underlying factors driving these sex-specific differences and consider the role of game type and context.

This study has several strengths. It included a large sample size with screen time data in early childhood and utilized linked provincial standardized testing data. The prospective design, spanning from 2008 to 2023, accounted for the year of testing, suggesting that the association between high levels of child screen time and academic achievement remained consistent despite societal changes and evolving screen-related technology, habits, and routines over time. Additionally, this study distinguished between different types of screen time, conducted separate analyses in male and female participants, and adjusted for multiple confounders.

### Limitations

This study has limitations. As an observational study examining associations, causality cannot be definitively assessed, despite the exposure being measured prior to the outcome. Likewise, residual confounding may also be present due to the observational design. The use of parent-reported questionnaires to measure child screen time may have introduced self-report and recall bias. However, given the young age of the participants, this method remains the most practical and common approach. We did not collect data on the content and context of child screen time, particularly regarding video games, which are important factors in screen time research. Social media use, an increasingly important area for policy, was not specifically examined in this study. While we measured handheld device use, which may include social media, we did not collect data on social media use over time. Another limitation is that TV, computer, and handheld device use were combined and collected as a single variable, which may introduce heterogeneity in the associations observed. Although the Ontario provincial standardized tests are objective and reliable measures of academic achievement that allow for year-to-year and province-wide comparability, we recognize their limitations, including possible teaching to the test and that they may not fully capture a child’s academic performance compared with teacher-assigned grades. Furthermore, the generalizability of our findings may be limited. The study population predominantly consisted of urban children recruited from primary care settings in the greater Toronto area. Our study sample was skewed toward higher socioeconomic status and had slightly higher scores on standardized tests compared with students across Ontario.^56^

## Conclusions

In this prospective cohort study of Canadian children recruited from primary care settings, high levels of early total screen time and TV and digital media time were associated with lower reading and math achievement in elementary school. Our findings underscore the importance of developing and testing targeted early guidelines and interventions to reduce screen time and TV and digital media exposure, with the goal of improving academic achievement in elementary school. Recommendations and interventions should be tailored to recognize that different types of screen time may have varying effects on male and female children, encouraging parents and educators to monitor and support healthy screen habits accordingly. Collaboration between health care professionals, schools, families, and policymakers is essential so that healthier screen use habits can be fostered early on to support development and later academic outcomes. While current screen time guidelines continue to recommend daily limits, they increasingly emphasize the importance of media quality and the context in which screens are used.^7^ This underscores the need to examine not only the duration of screen time, but also its content and context, such as the quality of content, school-based screen use, and the degree of family involvement. Future research could examine screen use comprehensively and explore how these various dimensions relate to academic achievement to inform more targeted and meaningful screen use recommendations.

## Notes

Supplement 1. **eTable 1.** Child Screen Time by Levels of Academic Achievement for Each Subject Area in Male Students **eTable 2.** Child Screen Time by Levels of Academic Achievement for Each Subject Area in Female Students

Supplement 2.
Nonauthor Collaborators

Supplement 3.
Data Sharing Statement
