# Supplementary material for: Screen Time and Standardized Academic Achievement Tests in Elementary School
Source: JAMA Netw Open. 2025 Oct 10;8(10):e2537092. doi: 10.1001/jamanetworkopen.2025.37092 (PMC12514615; doi:10.1001/jamanetworkopen.2025.37092)
Supplement: Supplement 1. — eTable 1. Child Screen Time by Levels of Academic Achievement for Each Subject Area in Male Students eTable 2. Child Screen Time by Levels of Academic Achievement for Each Subject Area in Female Students [file jamanetwopen-e2537092-s001.pdf]

Supplemental Online Content

Li X, Keown-Stoneman CD, Omand JA, et al. Screen time and standardized academic achievement tests in elementary school. *JAMA Netw Open*. 2025;8(10):e2537092. doi:10.1001/jamanetworkopen.2025.37092

eTable 1. Child Screen Time by Levels of Academic Achievement for Each Subject Area in Male Students

eTable 2. Child Screen Time by Levels of Academic Achievement for Each Subject Area in Female Students

This supplemental material has been provided by the authors to give readers additional information about their work.

© 2025 Li X et al. *JAMA Network Open*.

eTable 1. Child Screen Time by Levels of Academic Achievement for Each Subject Area in Male Students

	**Males with Grade 3 standardized test data, N=1714**			**Males with Grade 6 standardized test data, N=1070**		
	**Below**	**At**	**Above**	**Below**	**At**	**Above**
**Reading**	n=212	n=809	n=348	n=98	n=771	n=200
Total screen time, min/day, Mean (SD)	115.47 (98.96)	108.38 (84.44)	94.07 (76.19)	123.43 (89.49)	116.60 (83.03)	113.35 (88.93)
TV and digital media, min/day, Mean (SD)	103.22 (87.72)	96.51 (71.79)	85.43 (71.07)	112.53 (84.87)	100.60 (70.61)	94.32 (78.32)
Video game, min/day, Mean (SD)	11.21 (39.02)	10.54 (23.60)	8.52 (19.65)	10.90 (29.03)	15.15 (30.94)	16.49 (33.17)
Video game users, No. (%)	27.4%	30.6%	28.2%	32.7%	37.7%	41.0%
**Writing**	n=269	n=1036	n=63	n=120	n=715	n=232
Total screen time, min/day, Mean (SD)	113.24 (93.89)	104.72 (83.51)	92.90 (70.34)	121.89 (81.12)	117.49 (85.85)	111.42 (83.35)
TV and digital media, min/day, Mean (SD)	101.81 (84.58)	93.38 (71.97)	87.43 (68.34)	107.66 (74.72)	101.57 (75.98)	93.68 (64.87)
Video game, min/day, Mean (SD)	9.54 (22.54)	10.55 (27.08)	5.48 (12.36)	11.56 (23.78)	15.47 (30.24)	15.51 (37.10)
Video game users, No. (%)	27.9%	30.3%	20.6%	34.2%	38.6%	37.9%
**Math**	n=373	n=911	n=430	n=364	n=516	n=190
Total screen time, min/day, Mean (SD)	110.63 (95.46)	102.41 (79.35)	89.93 (71.11)	123.10 (88.06)	115.68 (82.03)	106.44 (84.60)
TV and digital media, min/day, Mean (SD)	99.50 (83.71)	92.33 (68.72)	80.23 (63.92)	106.49 (76.23)	99.92 (70.79)	90.50 (74.88)
Video game, min/day, Mean (SD)	9.92 (32.98)	9.18 (21.51)	9.29 (21.43)	15.14 (34.20)	15.34 (31.55)	13.77 (23.29)
Video game users, No. (%)	26.0%	28.4%	28.6%	34.3%	38.4%	43.2%

© 2025 Li X et al. *JAMA Network Open*.

eTable 2. Child Screen Time by Levels of Academic Achievement for Each Subject Area in Female Students

	Females with Grade 3 standardized test data, N=1608			Females with Grade 6 standardized test data, N=1014		
	**Below**	**At**	**Above**	**Below**	**At**	**Above**
**Reading**	n=124	n=640	n=457	n=42	n=657	n=315
Total screen time, min/day, Mean (SD)	110.80 (97.42)	99.89 (83.88)	86.12 (69.77)	124.66 (88.70)	106.06 (71.19)	96.54 (84.62)
TV and digital media, min/day, Mean (SD)	103.91 (91.04)	93.98 (75.50)	82.72 (66.20)	115.78 (85.30)	100.44 (66.31)	87.62 (72.31)
Video game, min/day, Mean (SD)	5.57 (15.74)	4.18 (14.68)	2.68 (11.75)	7.65 (16.06)	4.80 (17.31)	4.99 (17.09)
Video game users, No. (%)	19.4%	13.6%	10.5%	21.4%	15.2%	14.9%
**Writing**	n=145	n=963	n=113	n=43	n=552	n=419
Total screen time, min/day, Mean (SD)	106.37 (95.18)	96.14 (80.60)	79.80 (57.10)	115.02 (59.11)	108.10 (77.75)	97.16 (76.11)
TV and digital media, min/day, Mean (SD)	100.68 (89.73)	90.82 (73.37)	77.67 (55.55)	105.23 (51.78)	102.44 (72.49)	89.21 (66.02)
Video game, min/day, Mean (SD)	4.44 (14.60)	3.85 (14.14)	2.09 (8.86)	9.78 (20.12)	4.18 (13.60)	5.53 (20.69)
Video game users, No. (%)	13.8%	13.4%	8.8%	27.9%	14.9%	14.8%
**Math**	n=391	n=877	n=339	n=332	n=511	n=170
Total screen time, min/day, Mean (SD)	103.04 (90.48)	93.25 (76.29)	77.88 (69.90)	113.12 (78.79)	101.92 (77.09)	91.82 (68.48)
TV and digital media, min/day, Mean (SD)	96.56 (81.51)	88.36 (70.04)	75.32 (68.21)	107.57 (73.70)	93.28 (67.10)	88.21 (65.40)
Video game, min/day, Mean (SD)	4.91 (17.11)	3.75 (13.03)	2.03 (9.97)	5.15 (15.84)	5.60 (19.75)	2.79 (9.80)
Video game users, No. (%)	15.1%	13.2%	9.7%	16.6%	15.5%	12.9%

© 2025 Li X et al. *JAMA Network Open*.
