# Supplementary material for: Screen Time and Standardized Academic Achievement Tests in Elementary School
Source: JAMA Netw Open. 2025 Oct 10;8(10):e2537092. doi: 10.1001/jamanetworkopen.2025.37092 (PMC12514615; doi:10.1001/jamanetworkopen.2025.37092)
Supplement: Supplement 2. — Nonauthor Collaborators [file jamanetwopen-e2537092-s002.pdf]

Supplemental Online Content: Nonauthor Collaborators
*First name, last name, and suffix (if applicable) are required and will appear in PubMed.

***Group Name(s): Members of the TARGet Kids! Collaboration (note members may have additional affiliations)**

***First Name and Middle Initial(s)**	***Last Name**	***Suffix (eg, Jr, III)**	Academic Degrees	Institution	Location (city, state/province, country)	Role or Contribution, eg, chair, principal investigator	Group (if more than 1 Group listed in the byline) and/or Subgroup (eg, Steering Committee)
Catherine S.	Birken		MD, MSc	The Hospital for Sick Children	Toronto, Ontario, Canada	Co-Leads	TARGet Kids! Collaboration
Jonathon L.	Maguire		MD, MSc	Unity Health Toronto	Toronto, Ontario, Canada	Co-Leads	TARGet Kids! Collaboration
Christopher	Allen		MSc, PMP	Unity Health Toronto	Toronto, Ontario, Canada	Executive Committee	TARGet Kids! Collaboration
Laura N.	Anderson		PhD	McMaster University	Hamilton, Ontario, Canada	Executive Committee	TARGet Kids! Collaboration
Katherine	Cost		PhD	McMaster University	Hamilton, Ontario, Canada	Executive Committee	TARGet Kids! Collaboration
Danielle	D’Annunzio		BA, LLM, PMP	The Hospital for Sick Children	Toronto, Ontario, Canada	Executive Committee	TARGet Kids! Collaboration
Michelle	Goodman		MSc	Unity Health Toronto	Toronto, Ontario, Canada	Executive Committee	TARGet Kids! Collaboration
Jameela	Kassam		MSc	The Hospital for Sick Children	Toronto, Ontario, Canada	Executive Committee	TARGet Kids! Collaboration
Charles	Keown-Stoneman		PhD	Unity Health Toronto	Toronto, Ontario, Canada	Executive Committee	TARGet Kids! Collaboration
Michelle	Mitchell		BA	The Hospital for Sick Children	Toronto, Ontario, Canada	Executive Committee	TARGet Kids! Collaboration
Marc	Denzel-Nunez		HBSc	Unity Health Toronto	Toronto, Ontario, Canada	Executive Committee	TARGet Kids! Collaboration
Jessica A.	Omand		RD, PhD	Toronto Metropolitan University	Toronto, Ontario, Canada	Executive Committee	TARGet Kids! Collaboration
Mateenah	Roksandic		BSc	Unity Health Toronto	Toronto, Ontario, Canada	Executive Committee	TARGet Kids! Collaboration
Sharon	Thadani		MLA/T	Unity Health Toronto	Toronto, Ontario, Canada	Executive Committee	TARGet Kids! Collaboration

*First name, last name, and suffix (if applicable) are required and will appear in PubMed.

***First Name and Middle Initial(s)**	***Last Name**	***Suffix (eg, Jr, III)**	Academic Degrees	Institution	Location (city, state/province, country)	Role or Contribution, eg, chair, principal investigator	Group (if more than 1 Group listed in the byline) and/or Subgroup (eg, Steering Committee)
Nusrat	Zaffar		MBBS	Unity Health Toronto	Toronto, Ontario, Canada	Executive Committee	TARGet Kids! Collaboration
Mary	Aglipay		MSc	University of Toronto	Toronto, Ontario, Canada	Trainee	TARGet Kids! Collaboration
Imaan	Bayoumi		MD, MSc	Queen's University	Kingston, Ontario, Canada	Investigator	TARGet Kids! Collaboration
Cornelia M.	Borkhoff		PhD	The Hospital for Sick Children	Toronto, Ontario, Canada	Investigator	TARGet Kids! Collaboration
Rahul	Chanchlani		MD, MSc	McMaster University	Hamilton, Ontario, Canada	Investigator	TARGet Kids! Collaboration
Alice	Charach		MD, MSc	The Hospital for Sick Children	Toronto, Ontario, Canada	Investigator	TARGet Kids! Collaboration
Larissa	Chomka		BASc, BSc	University of Toronto	Toronto, Ontario, Canada	Trainee	TARGet Kids! Collaboration
Curtis	D'Hollander		RD, MSc	University of Toronto	Toronto, Ontario, Canada	Trainee	TARGet Kids! Collaboration
Anne	Fuller		MD, MSc	McMaster University	Hamilton, Ontario, Canada	Investigator	TARGet Kids! Collaboration
Benita	Hosseini		MSc, PhD	University of Toronto	Toronto, Ontario, Canada	Investigator	TARGet Kids! Collaboration
Kiki	Hudson		BHSc	McMaster University	Hamilton, Ontario, Canada	Trainee	TARGet Kids! Collaboration
Tatjana	Kay		MSc	Toronto Metropolitan University	Toronto, Ontario, Canada	Trainee	TARGet Kids! Collaboration
Sahar	Khademioore		MSc	McMaster University	Hamilton, Ontario, Canada	Trainee	TARGet Kids! Collaboration
Fatemeh	Khorramrouz		BSc, MSc	University of Toronto	Toronto, Ontario, Canada	Trainee	TARGet Kids! Collaboration
Laura	Kinlin		MD, MPH	The Hospital for Sick Children	Toronto, Ontario, Canada	Investigator	TARGet Kids! Collaboration

Supplemental Online Content: Nonauthor Collaborators
*First name, last name, and suffix (if applicable) are required and will appear in PubMed.

***First Name and Middle Initial(s)**	***Last Name**	***Suffix (eg, Jr, III)**	Academic Degrees	Institution	Location (city, state/province, country)	Role or Contribution, eg, chair, principal investigator	Group (if more than 1 Group listed in the byline) and/or Subgroup (eg, Steering Committee)
Michaela	Kucab		RD, MHSc	University of Toronto	Toronto, Ontario, Canada	Trainee	TARGet Kids! Collaboration
Sabrina	Lai		HBSc	University of Toronto	Toronto, Ontario, Canada	Trainee	TARGet Kids! Collaboration
Rosella	Le		BSc, PMDip	University of Toronto	Toronto, Ontario, Canada	Trainee	TARGet Kids! Collaboration
Patricia	Li		MD, MSc	McGill University	Montreal, Quebec, Canada	Investigator	TARGet Kids! Collaboration
Paraskevi	Massara		PhD	University of Toronto	Toronto, Ontario, Canada	Trainee	TARGet Kids! Collaboration
Pat	Parkin		MD	The Hospital for Sick Children	Toronto, Ontario, Canada	Investigator	TARGet Kids! Collaboration
Nav	Persaud		MD, MSc	Unity Health Toronto	Toronto, Ontario, Canada	Investigator	TARGet Kids! Collaboration
Sarah	Rae		BHSc, MSc	University of Toronto	Toronto, Ontario, Canada	Trainee	TARGet Kids! Collaboration
Katia	Sinopoli		PhD	The Hospital for Sick Children	Toronto, Ontario, Canada	Investigator	TARGet Kids! Collaboration
Kevin	Thorpe		MMath	University of Toronto	Toronto, Ontario, Canada	Investigator	TARGet Kids! Collaboration
Leigh	Vanderloo		PhD	ParticipAction	Toronto, Ontario, Canada	Investigator	TARGet Kids! Collaboration
Peter	Wong		MBBS, MPH, PhD	The Hospital for Sick Children	Toronto, Ontario, Canada	Investigator	TARGet Kids! Collaboration
Karen	Zhang		BSc	University of Toronto	Toronto, Ontario, Canada	Trainee	TARGet Kids! Collaboration
Piyumi K.	Mudiyanselage		MSc	The Hospital for Sick Children	Toronto, Ontario, Canada	Research Staff	TARGet Kids! Collaboration
Xuedi	Li		MSc	The Hospital for Sick Children	Toronto, Ontario, Canada	Research Staff	TARGet Kids! Collaboration

Supplemental Online Content: Nonauthor Collaborators
*First name, last name, and suffix (if applicable) are required and will appear in PubMed.

***First Name and Middle Initial(s)**	***Last Name**	***Suffix (eg, Jr, III)**	Academic Degrees	Institution	Location (city, state/province, country)	Role or Contribution, eg, chair, principal investigator	Group (if more than 1 Group listed in the byline) and/or Subgroup (eg, Steering Committee)
Carol	Lee		RN, BScN	The Hospital for Sick Children	Toronto, Ontario, Canada	Research Staff	TARGet Kids! Collaboration
Yulika	Yoshida-Montezuma		MPH	The Hospital for Sick Children	Toronto, Ontario, Canada	Research Staff	TARGet Kids! Collaboration
Durdana	Akhter		CCRP	The Hospital for Sick Children	Toronto, Ontario, Canada	Clinical Site Research Staff	TARGet Kids! Collaboration
Trinelle	Brown		BHSc	Unity Health Toronto	Toronto, Ontario, Canada	Clinical Site Research Staff	TARGet Kids! Collaboration
Pamela R.	Flores		LMCC	Unity Health Toronto	Toronto, Ontario, Canada	Clinical Site Research Staff	TARGet Kids! Collaboration
Ofri	Hayosh		MD, IBCLC	Unity Health Toronto	Toronto, Ontario, Canada	Clinical Site Research Staff	TARGet Kids! Collaboration
Maleeha	Hussain		CSMLS/ML PAO	Unity Health Toronto	Toronto, Ontario, Canada	Clinical Site Research Staff	TARGet Kids! Collaboration
Sangeetha	Loganathan		BPT	The Hospital for Sick Children	Toronto, Ontario, Canada	Clinical Site Research Staff	TARGet Kids! Collaboration
Kajalben	Shah		BHMS, OCGC	The Hospital for Sick Children	Toronto, Ontario, Canada	Clinical Site Research Staff	TARGet Kids! Collaboration
Laurie	Thompson		MLT	The Hospital for Sick Children	Toronto, Ontario, Canada	Clinical Site Research Staff	TARGet Kids! Collaboration
Jennifer	Batten			TARGet Kids! Parent and Clinician Team	Toronto, Ontario, Canada	Parent Partner	TARGet Kids! Collaboration
Jennifer	Chan			TARGet Kids! Parent and Clinician Team	Toronto, Ontario, Canada	Parent Partner	TARGet Kids! Collaboration
Kim	De Castris-Garcia			TARGet Kids! Parent and Clinician Team	Toronto, Ontario, Canada	Parent Partner	TARGet Kids! Collaboration
Maureen	Colford			TARGet Kids! Parent and Clinician Team	Toronto, Ontario, Canada	Parent Partner	TARGet Kids! Collaboration
Sharon	Dharman			TARGet Kids! Parent and Clinician Team	Toronto, Ontario, Canada	Parent Partner	TARGet Kids! Collaboration

*First name, last name, and suffix (if applicable) are required and will appear in PubMed.

***First Name and Middle Initial(s)**	***Last Name**	***Suffix (eg, Jr, III)**	Academic Degrees	Institution	Location (city, state/province, country)	Role or Contribution, eg, chair, principal investigator	Group (if more than 1 Group listed in the byline) and/or Subgroup (eg, Steering Committee)
Sarah	Kelleher			TARGet Kids! Parent and Clinician Team	Toronto, Ontario, Canada	Parent Partner	TARGet Kids! Collaboration
Salimah	Nasser			TARGet Kids! Parent and Clinician Team	Toronto, Ontario, Canada	Parent Partner	TARGet Kids! Collaboration
Tammara	Pabon			TARGet Kids! Parent and Clinician Team	Toronto, Ontario, Canada	Parent Partner	TARGet Kids! Collaboration
Michelle	Rhodes			TARGet Kids! Parent and Clinician Team	Toronto, Ontario, Canada	Parent Partner	TARGet Kids! Collaboration
Rafael	Salsa			TARGet Kids! Parent and Clinician Team	Toronto, Ontario, Canada	Parent Partner	TARGet Kids! Collaboration
Julie	Skelding			TARGet Kids! Parent and Clinician Team	Toronto, Ontario, Canada	Parent Partner	TARGet Kids! Collaboration
Daniel	Stern			TARGet Kids! Parent and Clinician Team	Toronto, Ontario, Canada	Parent Partner	TARGet Kids! Collaboration
Kerry	Stewart			TARGet Kids! Parent and Clinician Team	Toronto, Ontario, Canada	Parent Partner	TARGet Kids! Collaboration
Erika S.	Tavares			TARGet Kids! Parent and Clinician Team	Toronto, Ontario, Canada	Parent Partner	TARGet Kids! Collaboration
Shannon	Weir-Seeley			TARGet Kids! Parent and Clinician Team	Toronto, Ontario, Canada	Parent Partner	TARGet Kids! Collaboration
Maria	Zaccaria-Cho			TARGet Kids! Parent and Clinician Team	Toronto, Ontario, Canada	Parent Partner	TARGet Kids! Collaboration
Ashan	Ameer			The Hospital for Sick Children	Toronto, Ontario, Canada	Youth Researcher	TARGet Kids! Collaboration
Isolde	Ardies			The Hospital for Sick Children	Toronto, Ontario, Canada	Youth Researcher	TARGet Kids! Collaboration
Soliana	Lijiam			The Hospital for Sick Children	Toronto, Ontario, Canada	Youth Researcher	TARGet Kids! Collaboration
William B.	Mazin			The Hospital for Sick Children	Toronto, Ontario, Canada	Youth Researcher	TARGet Kids! Collaboration

Supplemental Online Content: Nonauthor Collaborators
*First name, last name, and suffix (if applicable) are required and will appear in PubMed.

***First Name and Middle Initial(s)**	***Last Name**	***Suffix (eg, Jr, III)**	Academic Degrees	Institution	Location (city, state/province, country)	Role or Contribution, eg, chair, principal investigator	Group (if more than 1 Group listed in the byline) and/or Subgroup (eg, Steering Committee)
Ariella	Poliszuk			The Hospital for Sick Children	Toronto, Ontario, Canada	Youth Researcher	TARGet Kids! Collaboration
Tahira	Rajwani			The Hospital for Sick Children	Toronto, Ontario, Canada	Youth Researcher	TARGet Kids! Collaboration
Netra	Senthil			The Hospital for Sick Children	Toronto, Ontario, Canada	Youth Researcher	TARGet Kids! Collaboration
Reena	Wu			The Hospital for Sick Children	Toronto, Ontario, Canada	Youth Researcher	TARGet Kids! Collaboration
Eric	Duku		PhD	McMaster University	Hamilton, Ontario, Canada	Offord Centre for Child Studies Collaboration	TARGet Kids! Collaboration
Magdalena	Janus		PhD	McMaster University	Hamilton, Ontario, Canada	Offord Centre for Child Studies Collaboration	TARGet Kids! Collaboration
Amanda	Offord		MSc	McMaster University	Hamilton, Ontario, Canada	Offord Centre for Child Studies Collaboration	TARGet Kids! Collaboration
Patricia	Raso		MSc	McMaster University	Hamilton, Ontario, Canada	Offord Centre for Child Studies Collaboration	TARGet Kids! Collaboration
Caroline	Reid-Westoby		PhD	McMaster University	Hamilton, Ontario, Canada	Offord Centre for Child Studies Collaboration	TARGet Kids! Collaboration
Emy	Abraham		MD	Pediatrics @ Humber College	Etobicoke, Ontario, Canada	Site Investigators (clinic practice)	TARGet Kids! Collaboration
Farah	Ali		MD	St. Michael's Hospital	Toronto, Ontario, Canada	Site Investigators (clinic practice)	TARGet Kids! Collaboration
Jilian	Baker		MD	St. Michael's Hospital	Toronto, Ontario, Canada	Site Investigators (clinic practice)	TARGet Kids! Collaboration
Hailey	Barootes		MD	St. Michael's Hospital	Toronto, Ontario, Canada	Site Investigators (clinic practice)	TARGet Kids! Collaboration

Supplemental Online Content: Nonauthor Collaborators
*First name, last name, and suffix (if applicable) are required and will appear in PubMed.

***First Name and Middle Initial(s)**	***Last Name**	***Suffix (eg, Jr, III)**	Academic Degrees	Institution	Location (city, state/province, country)	Role or Contribution, eg, chair, principal investigator	Group (if more than 1 Group listed in the byline) and/or Subgroup (eg, Steering Committee)
Tony	Barozzino		MD	St. Michael's Hospital	Toronto, Ontario, Canada	Site Investigators (clinic practice)	TARGet Kids! Collaboration
Sylvie	Bergeron		MD	Pediatrics @ Humber College	Etobicoke, Ontario, Canada	Site Investigators (clinic practice)	TARGet Kids! Collaboration
Melanie	Beswick		MD	St. Michael's Hospital	Toronto, Ontario, Canada	Site Investigators (clinic practice)	TARGet Kids! Collaboration
Seema	Bhandarkar		RN	St. Michael's Hospital	Toronto, Ontario, Canada	Site Investigators (clinic practice)	TARGet Kids! Collaboration
Tali	Bogler		MD	St. Michael's Hospital	Toronto, Ontario, Canada	Site Investigators (clinic practice)	TARGet Kids! Collaboration
Caroline	Calpin		MD	Westway Children's Clinic	Etobicoke, Ontario, Canada	Site Investigators (clinic practice)	TARGet Kids! Collaboration
Douglas	Campbell		MD	St. Michael's Hospital	Toronto, Ontario, Canada	Site Investigators (clinic practice)	TARGet Kids! Collaboration
Sohail	Cheema		MD	St. Michael's Hospital	Toronto, Ontario, Canada	Site Investigators (clinic practice)	TARGet Kids! Collaboration
Brian	Chisamore		MD	Village Park Pediatrics	Toronto, Ontario, Canada	Site Investigators (clinic practice)	TARGet Kids! Collaboration
Justine	Cohen-Silver		MD	St. Michael's Hospital	Toronto, Ontario, Canada	Site Investigators (clinic practice)	TARGet Kids! Collaboration
Karoon	Danayan		MD	Danforth Pediatrics	Toronto, Ontario, Canada	Site Investigators (clinic practice)	TARGet Kids! Collaboration
Anh	Do		MD	Pediatric Experience	Toronto, Ontario, Canada	Site Investigators (clinic practice)	TARGet Kids! Collaboration
Michelle	Durst		MD	St. Michael's Hospital	Toronto, Ontario, Canada	Site Investigators (clinic practice)	TARGet Kids! Collaboration
Allison	Farber		MD	St. Michael's Hospital	Toronto, Ontario, Canada	Site Investigators (clinic practice)	TARGet Kids! Collaboration
Sloane	Freeman		MD	St. Michael's Hospital	Toronto, Ontario, Canada	Site Investigators (clinic practice)	TARGet Kids! Collaboration

Supplemental Online Content: Nonauthor Collaborators
*First name, last name, and suffix (if applicable) are required and will appear in PubMed.

***First Name and Middle Initial(s)**	***Last Name**	***Suffix (eg, Jr, III)**	Academic Degrees	Institution	Location (city, state/province, country)	Role or Contribution, eg, chair, principal investigator	Group (if more than 1 Group listed in the byline) and/or Subgroup (eg, Steering Committee)
Ryan	Giroux		MD	St. Michael's Hospital	Toronto, Ontario, Canada	Site Investigators (clinic practice)	TARGet Kids! Collaboration
Archana	Gupta		MD, PhD	St. Michael's Hospital	Toronto, Ontario, Canada	Site Investigators (clinic practice)	TARGet Kids! Collaboration
Leah D.	Harrington		MD	Westway Children's Clinic	Etobicoke, Ontario, Canada	Site Investigators (clinic practice)	TARGet Kids! Collaboration
Karl	Igar		MD	St. Michael's Hospital	Toronto, Ontario, Canada	Site Investigators (clinic practice)	TARGet Kids! Collaboration
Emma	Jeavons		MD	St. Michael's Hospital	Toronto, Ontario, Canada	Site Investigators (clinic practice)	TARGet Kids! Collaboration
Puneeta	Khurana		MD	St. Michael's Hospital	Toronto, Ontario, Canada	Site Investigators (clinic practice)	TARGet Kids! Collaboration
Eddy	Lau		MD	Village Park Pediatrics	Toronto, Ontario, Canada	Site Investigators (clinic practice)	TARGet Kids! Collaboration
Renata	Leong		MD	St. Michael's Hospital	Toronto, Ontario, Canada	Site Investigators (clinic practice)	TARGet Kids! Collaboration
Jennifer	McCabe		MD	St. Michael's Hospital	Toronto, Ontario, Canada	Site Investigators (clinic practice)	TARGet Kids! Collaboration
Aleks	Meret		MD	Danforth Pediatrics	Toronto, Ontario, Canada	Site Investigators (clinic practice)	TARGet Kids! Collaboration
Annette	Miller		MD	One Care	Scarborough, Ontario,Canada	Site Investigators (clinic practice)	TARGet Kids! Collaboration
Sharon	Mintz		MD	St. Michael's Hospital	Toronto, Ontario, Canada	Site Investigators (clinic practice)	TARGet Kids! Collaboration
Rosemary	Moodie		MD	Pediatrics @ Humber College	Etobicoke, Ontario, Canada	Site Investigators (clinic practice)	TARGet Kids! Collaboration
Navyn	Naran		MD	One Care	Scarborough, Ontario,Canada	Site Investigators (clinic practice)	TARGet Kids! Collaboration
Diedre	O'Sullivan		MD	St. Michael's Hospital	Toronto, Ontario, Canada	Site Investigators (clinic practice)	TARGet Kids! Collaboration

Supplemental Online Content: Nonauthor Collaborators
*First name, last name, and suffix (if applicable) are required and will appear in PubMed.

***First Name and Middle Initial(s)**	***Last Name**	***Suffix (eg, Jr, III)**	Academic Degrees	Institution	Location (city, state/province, country)	Role or Contribution, eg, chair, principal investigator	Group (if more than 1 Group listed in the byline) and/or Subgroup (eg, Steering Committee)
Sureka	Paramaguru		MD	One Care	Scarborough, Ontario,Canada	Site Investigators (clinic practice)	TARGet Kids! Collaboration
Eliza	Pope		MD	Pediatric Experience	Toronto, Ontario, Canada	Site Investigators (clinic practice)	TARGet Kids! Collaboration
Michelle	Porepa		MD	Pediatric Experience	Toronto, Ontario, Canada	Site Investigators (clinic practice)	TARGet Kids! Collaboration
Julia	Rackal		MD	St. Michael's Hospital	Toronto, Ontario, Canada	Site Investigators (clinic practice)	TARGet Kids! Collaboration
Evelyn	Rozenblyum		MD	St. Michael's Hospital	Toronto, Ontario, Canada	Site Investigators (clinic practice)	TARGet Kids! Collaboration
Lorna	Sampson		MD	Pediatric Experience	Toronto, Ontario, Canada	Site Investigators (clinic practice)	TARGet Kids! Collaboration
Manjot	Sarao		MD	St. Michael's Hospital	Toronto, Ontario, Canada	Site Investigators (clinic practice)	TARGet Kids! Collaboration
Janet	Saunderson		MD	Pediatric Experience	Toronto, Ontario, Canada	Site Investigators (clinic practice)	TARGet Kids! Collaboration
Michael	Sgro		MD	St. Michael's Hospital	Toronto, Ontario, Canada	Site Investigators (clinic practice)	TARGet Kids! Collaboration
Desmond	She		MD	Village Park Pediatrics	Toronto, Ontario, Canada	Site Investigators (clinic practice)	TARGet Kids! Collaboration
Valene	Singh		MD	St. Michael's Hospital	Toronto, Ontario, Canada	Site Investigators (clinic practice)	TARGet Kids! Collaboration
Chantal	Sorhaindo		MN, NP(PHC-GH), RN-EC	St. Michael's Hospital	Toronto, Ontario, Canada	Site Investigators (clinic practice)	TARGet Kids! Collaboration
Karen	Swirsky		MD	St. Michael's Hospital	Toronto, Ontario, Canada	Site Investigators (clinic practice)	TARGet Kids! Collaboration
Mathura	Thevarajah		MD	Danforth Pediatrics	Toronto, Ontario, Canada	Site Investigators (clinic practice)	TARGet Kids! Collaboration

Supplemental Online Content: Nonauthor Collaborators
*First name, last name, and suffix (if applicable) are required and will appear in PubMed.

***First Name and Middle Initial(s)**	***Last Name**	***Suffix (eg, Jr, III)**	Academic Degrees	Institution	Location (city, state/province, country)	Role or Contribution, eg, chair, principal investigator	Group (if more than 1 Group listed in the byline) and/or Subgroup (eg, Steering Committee)
Philip	Tsang		MD	St. Michael's Hospital	Toronto, Ontario, Canada	Site Investigators (clinic practice)	TARGet Kids! Collaboration
Subrata	Verma		MD	One Care	Scarborough, Ontario,Canada	Site Investigators (clinic practice)	TARGet Kids! Collaboration
Cheng T	Wang		MD	St. Michael's Hospital	Toronto, Ontario, Canada	Site Investigators (clinic practice)	TARGet Kids! Collaboration
Karen	Weyman		MD	St. Michael's Hospital	Toronto, Ontario, Canada	Site Investigators (clinic practice)	TARGet Kids! Collaboration
Patricia	Windrim		MD	St. Michael's Hospital	Toronto, Ontario, Canada	Site Investigators (clinic practice)	TARGet Kids! Collaboration
Peter	Wong		MD	Pediatrics @ Humber College	Etobicoke, Ontario, Canada	Site Investigators (clinic practice)	TARGet Kids! Collaboration
Jonathan	Wong		MD	Pediatrics @ Humber College	Etobicoke, Ontario, Canada	Site Investigators (clinic practice)	TARGet Kids! Collaboration
Dongning	Wu		MD	One Care	Scarborough, Ontario,Canada	Site Investigators (clinic practice)	TARGet Kids! Collaboration
Ethel	Ying		MD	St. Michael's Hospital	Toronto, Ontario, Canada	Site Investigators (clinic practice)	TARGet Kids! Collaboration
Elizabeth	Young		MD	St. Michael's Hospital	Toronto, Ontario, Canada	Site Investigators (clinic practice)	TARGet Kids! Collaboration
Esmot A.	Begum		PhD	Unity Health Toronto	Toronto, Ontario, Canada	Applied Health Research Centre	TARGet Kids! Collaboration
Marzena	Boczulak		HBSc	Unity Health Toronto	Toronto, Ontario, Canada	Applied Health Research Centre	TARGet Kids! Collaboration
Nilifa	Desilva		MPH	Unity Health Toronto	Toronto, Ontario, Canada	Applied Health Research Centre	TARGet Kids! Collaboration
Gurpreet	Lakhanpal		MSc, CCRP, PMP	Unity Health Toronto	Toronto, Ontario, Canada	Applied Health Research Centre	TARGet Kids! Collaboration
Hamed	Moazami		MSc	Unity Health Toronto	Toronto, Ontario, Canada	Applied Health Research Centre	TARGet Kids! Collaboration

Supplemental Online Content: Nonauthor Collaborators
*First name, last name, and suffix (if applicable) are required and will appear in PubMed.

***First Name and Middle Initial(s)**	***Last Name**	***Suffix (eg, Jr, III)**	Academic Degrees	Institution	Location (city, state/province, country)	Role or Contribution, eg, chair, principal investigator	Group (if more than 1 Group listed in the byline) and/or Subgroup (eg, Steering Committee)
George S.	Charames		PhD, FACMG	Mount Sinai Hospital	Toronto, Ontario, Canada	Mount Sinai Services Laboratory	TARGet Kids! Collaboration
Andrea	Djolovic		MSc, CCGC	Mount Sinai Hospital	Toronto, Ontario, Canada	Mount Sinai Services Laboratory	TARGet Kids! Collaboration
Chelsea	Gorscak-Dunn			Mount Sinai Hospital	Toronto, Ontario, Canada	Mount Sinai Services Laboratory	TARGet Kids! Collaboration
Reenu	Joseph		MSc	Mount Sinai Hospital	Toronto, Ontario, Canada	Mount Sinai Services Laboratory	TARGet Kids! Collaboration
Melanie	Peralta		MLT	Mount Sinai Hospital	Toronto, Ontario, Canada	Mount Sinai Services Laboratory	TARGet Kids! Collaboration
