# Supplementary material for: Screen Time and Standardized Academic Achievement Tests in Elementary School
Source: JAMA Netw Open. 2025 Oct 10;8(10):e2537092. doi: 10.1001/jamanetworkopen.2025.37092 (PMC12514615; doi:10.1001/jamanetworkopen.2025.37092)
Supplement: Supplement 3. — Data Sharing Statement [file jamanetwopen-e2537092-s003.pdf]

Data Sharing Statement

Li. Screen Time and Standardized Academic Achievement Tests in Elementary School. *JAMA Netw Open*. Published October 10, 2025. doi:10.1001/jamanetworkopen.2025.37092

Data
Data available: No

Additional Information
Explanation for why data not available: The full data are not freely available to respect the confidentiality of our participants, ensure data integrity, and avoid scientific overlap between projects. Data may be available upon request by contacting www.targetkids.ca/contact-us/. Once initial contact has been made, we request a short research proposal which will be subject to review by the TARGet Kids! Scientific Committee and approval by institutional REBs.
